# Crosstalk between tumor-associated macrophages and tumor cells promotes chemoresistance via CXCL5/PI3K/AKT/mTOR pathway in gastric cancer

**DOI:** 10.1186/s12935-022-02717-5

**Published:** 2022-09-23

**Authors:** Pengfei Su, Lin Jiang, Yingjing Zhang, Tian Yu, Weiming Kang, Yuqin Liu, Jianchun Yu

**Affiliations:** 1 Department of General Surgery, Peking Union Medical College Hospital, Chinese Academy of Medical Sciences and Peking Union Medical College, Beijing, 100730 China; 2 Graduate School, Chinese Academy of Medical Sciences and Peking Union Medical College, Beijing, 100005 China; 3 Department of Pathology, Institute of Basic Medical Sciences, Chinese Academy of Medical Sciences and Peking Union Medical College, Beijing, 100005 China

**Keywords:** Gastric cancer, Tumor-associated macrophage, Tumor microenvironment, Chemoresistance, CXCL5

## Abstract

**Background:**

5-fluorouracil (5-FU)-based chemotherapy regimen has been widely used for the treatment of gastric cancer, but meanwhile the development of chemotherapeutic resistance remains a major clinical challenge. Tumor microenvironment (TME) frequently correlates with the development of chemoresistance in human cancer. As a major component of TME, the role of tumor-associated macrophages (TAMs) in the chemoresistance of gastric cancer has not been fully elucidated.

**Methods:**

Immunohistochemistry (IHC) was applied to detect the density of TAMs in clinical samples of 103 patients with gastric cancer who had undergone 5-FU-based neoadjuvant chemotherapy. 5-FU-resistant gastric cell lines MKN45-R and HGC27-R were established, macrophages were then separately co-cultured with MKN45-R, HGC27-R cells and their parental cells. The effect of gastric cancer cells on the polarization of macrophages, the biological function of M2-polaried macrophages and the mechanism for promoting 5-FU-resistance were investigated. Then the correlation between the expression of CXC motif chemokine ligand 5 (CXCL5) and the infiltration of hemoglobin scavenger receptor (CD163) positive and mannose receptor (CD206) positive macrophages was analyzed, the prognostic value of CXCL5 expression in clinical samples was further explored.

**Results:**

The high infiltration of macrophages marked by CD68 in gastric cancer samples was significantly associated with the resistance of gastric cancer to chemotherapy. Gastric cancer cells could modulate macrophages to M2-like polarization through indirect co-culture, and chemoresistant cells were more efficient in inducing macrophages polarization to M2 phenotype. Co-culturing M2-polarized macrophages in turn enhanced 5-FU-resistance of gastric cancer cells, and it was further verified that CXCL5 derived from M2-polarized macrophages promoted chemoresistance through activing the PI3K/AKT/mTOR pathway. Besides, high level of CXCL5 could recruit monocytes to form more M2-polarized macrophages. Clinically, high expression of CXCL5 in gastric cancer samples was associated with the high infiltration of CD163 positive macrophages and CD206 positive macrophages, and patients with high expression of CXCL5 presented lower overall survival (OS) rates than those with low expression of CXCL5.

**Conclusion:**

Interaction between TAMs and gastric cancer cells promoted chemoresistance in gastric cancer via CXCL5/PI3K/AKT/mTOR pathway. Thus, targeting TAMs and blocking the cell–cell crosstalk between TAMs and gastric cancer cells may represent prospective therapeutic strategies for patients with gastric cancer.

**Supplementary Information:**

The online version contains supplementary material available at 10.1186/s12935-022-02717-5.

## Background

Gastric cancer is one of the most prevalent malignancies, accounting for the second leading cause of cancer-related mortality worldwide [1]. With the development of surgical technique, the prognosis of early gastric cancer has improved. Nevertheless, due to the low rate of early diagnosis, the majority of patients are diagnosis with advanced gastric cancer, for which chemotherapy is one of the major therapeutic strategies [2]. Even though combined chemotherapy before and after surgical operation has been proved to increase patients’ survival rates, the development of chemoresistance is still a major obstacle to obtaining effective chemotherapy [3]. 5-fluorouracil (5-FU) remains to be the first-line chemotherapeutic drug for gastric cancer, however, chemoresistance usually occurs with unsatisfactory clinical outcomes. Therefore, a better understanding of molecular mechanism to 5-FU resistance is critical for improving the clinical outcome of gastric cancer.

As the internal environment where tumor cells form and live, tumor microenvironment (TME) consists of not only tumor cells but also various nonmalignant stromal cells and extracellular matrix those participate in the progression of tumor [4]. In addition, studies have demonstrated that the crosstalk between tumor cells and other components of TME facilitates the development of chemoresistance [5–7]. Macrophages that infiltrate in the malignant tumor are defined as tumor-associated macrophages (TAMs), which constitute the dominant immune cells in TME and have been found to play a critical role in tumor progression [8, 9]. TAMs are heterogeneous cells and can be broadly classified into the classically activated phenotype (M1) and the alternatively activated phenotype (M2) depending on their distinct microenvironmental stimulating signals [10, 11]. In most solid tumors, M1 macrophages exhibit anti-tumor effect, expressing specific M1 markers like CD86 and CD80 and secreting cytokines such as interleukin (IL)6, IL12 and tumor necrosis factor (TNF)α, whereas M2 macrophages can support the malignant progression of tumor, expressing CD163, CD204 and CD206 and secreting IL4, IL10, vascular endothelial growth factor (VEGF) and arginase (Arg)-1 [4, 8, 12, 13]. Despite the phenotypic diversity, TAMs often present the M2-like phenotype with the progression of tumor, expressing characteristic markers such as the mannose receptor (CD206) and hemoglobin scavenger receptor (CD163) and correlating with poor prognosis in several solid tumors [8, 14, 15]. Increasing evidence has indicated that TAMs can mediate the chemoresistance of several malignant tumors, and targeting TAMs was considered to be a promising combinational therapy for cancer treatment [5, 16–18]. However, the role of TAMs in the development of chemoresistance in gastric cancer has not been elucidated so far. Thus, research on the reciprocal interaction between TAMs and gastric cancer cells might provide a novel perspective for the mechanism of chemoresistance.

TAMs secrete a variety of cytokines and chemokines into the TME and these small proteins are important modulators that could promote the development of therapeutic resistance. CC-chemokine ligand 2 (CCL2) secreted by TAMs was revealed to activate the PI3K/AKT/mTOR pathway in breast cancer cells, thus induced resistance to tamoxifen treatment in breast cancer [16]. TAMs regulated 5-FU-mediated colorectal cancer chemoresistance via the EMT program and caspase-mediated apoptosis by releasing CCL22 [17]. Macrophage-derived IL-6 was found to confer chemoresistance in colorectal cancer by regulating the IL-6R/STAT3/miR-204-5p axis [19]. Based on the above research status, it was speculated that cytokines or chemokines secreted from TAMs might promote the development of chemoresistance in gastric cancer.

In the present study, we aimed to explore the interaction between TAMs and the chemoresistant phenotype of gastric cancer cells. We first explored the clinical value of the CD68 (TAMs marker) in gastric cancer tissues from patients with gastric cancer who had undergone 5-FU-based neoadjuvant chemotherapy and elucidated the correlation between the infiltration of TAMs and the resistance of gastric cancer to chemotherapy. Then we found that 5-FU-resistant gastric cancer cells could effectively induce macrophages to polarize to M2 phenotype, which in turn promoted 5-FU-resistance in gastric cancer cells. We also identified a specific chemokine, CXC motif chemokine ligand 5 (CXCL5), derived from TAMs to promote 5-FU-resistance of gastric cancer cells and further investigated the underlying molecular mechanism. Moreover, immunohistochemistry was carried out on tumor samples to examine the correlation between CXCL5 expression and disease prognosis. Our findings delineated the interaction between TAMs and gastric cancers cells, improved the understanding of how TAMs promoted chemoresistance of gastric cancer, and might provide a novel therapeutic strategy for patients with chemoresistant gastric cancer.

## Materials and methods

### Collection of clinical samples

Paraffin-embedded samples of primary lesions from 103 patients with gastric cancer who had undergone 5-FU based neoadjuvant chemotherapy prior to radical resection at Peking Union Medical College Hospital between 2015 and 2017 were used. Patients were divided into two groups according to the evaluation of pathological response based on the guidelines of College of American Pathologists (CAP) [20]. CAP 0, CAP 1 and CAP 2 were defined as pathological response whereas CAP 3 was defined as no pathological response. 67 patients were elected to pathological response group and 36 patients were elected to no pathological response group for further research. Clinical samples were gathered with written informed consent of patients according to a protocol reviewed and approved by the Institutional Review Board of Peking Union Medical College Hospital.

### Immunohistochemistry (IHC)

A total of 103 archived clinical samples were fixed in 10% formaldehyde solution, embedded in paraffin and serially severed into 4 μm sections. After deparaffinized in xylene and rehydrated in graded ethanol, microwave heating with sodium citrate retrieval buffer (pH 6.0) was performed for antigen retrieval. The endogenous peroxidase was inactivated by treatment with 3% H_2_O_2_ for 10 min. Tissue sections were incubated with blocking buffer followed by incubation with primary antibodies Anti-CD68 (1:100, Cell Signaling Technology, MA, USA), Anti-CD163 (1:100, Cell Signaling Technology, MA, USA), Anti-CD206 (1:100, Cell Signaling Technology, MA, USA) and Anti-CXCL5 (1:200, Abcam, Cambridge, UK) at 4 °C overnight. After washing with PBS, the secondary antibody horseradish peroxidase (HRP)-conjugated Anti-Rabbit IgG (1:100, Cell Signaling Technology, MA, USA) was added for 30 min’ incubation at room temperature. 3, 3ʹ-diaminobenzidine (DAB) regent was applied for visualizing staining subsequent to PBS washing, then all slices were re-dyed with hematoxylin, dehydrated and sealed for microscopic examination. At least three slices were taken from each tumor tissue and five independent fields were randomly selected from each slice for detection. CXCL5 immunoreactivity was scored by multiplying the staining percentage scores (~ 5% scores 0; 5% ~ 25% scores 1; 25% ~ 50% scores 2; 50% ~ 75% scores 3; 75% ~ 100% scores 4) and staining intensity scores (0, no staining; 1, weak; 2, moderate; 3, strong). A final score of 0–3 was defined as low expression, while others were defined as high expression. CD68, CD163 and CD206 immunoreactivity were analyzed by calculating the mean number of positive cells in 5 random 400-fold fields. Two independent pathologists observed and scored the slices without knowledge of the patients’ clinical information.

### Cell lines and cell culture

Human gastric cancer cell lines MKN45 and HGC27, and human mononuclear cells (THP-1) were acquired from the Cell Resource Center of Peking Union Medical College (Beijing, China). The cells were maintained in RPMI-1640 medium (Gibco, Carlsbad, CA, USA) incorporating 10% fetal bovine serum (FBS) (Gibco, Carlsbad, CA, USA) and 1% penicillin/streptomycin (Gibco, Carlsbad, CA, USA) in a humidified 37 °C incubator with 5% CO_2_. 0.25% trypsin (NCM Biotech, Suzhou, Jiangsu, China) was administered in the logarithmic growth phase for cell digestion and passage.

Gastric cancer cell lines were regarded as 5-FU-sensitive (MKN45-S and HGC27-S) and the IC_50_ of 5-FU was detected. 5-FU-resistent cell lines (MKN45-R and HGC27-R) were generated by repetitively exposing gastric cancer cells to increasing concentrations of 5-FU over a 10 month period and the acquired 5-FU resistance was confirmed by detecting the IC_50_ of 5-FU and resistance index. THP-1 monocytes were differentiated into macrophages by 24 h incubation with 100 ng/ml phorbol 12-myristate 13-acetate (PMA) (Sigma-Aldrich, St.louis, MO, USA) for 24 h. Adherent cells were washed twice with culture medium followed by 24 h incubation to obtain the resting state of macrophages (M0). Method of detaching PMA-treated THP-1 cells from the culture dish was demonstrated in Additional file 1: Text S1.

### Co-culture of cancer cells and macrophages

Transwell chambers (6-well plates, 0.4-μm pore size; Corning, NY, USA) were used for co-culture. MKN45-S, HGC27-S, MKN45-R and HGC27-R cells were seeded onto the upper chambers, and M0 macrophages were placed in the lower chambers. After 48 h of co-culture, TAMs from 5-FU-sensitive TME (MS) and 5-FU-resistant TME (MR) were obtained and harvested for experimental analysis. To investigate the effect of TAMs with different phenotypes on gastric cancer cells, MS and MR were transferred to the upper chambers and gastric cancer cells were placed in the lower chambers for 48 h of co-culture.

### Cell counting kit-8 (CCK-8) assay

A cell counting kit-8 (CCK-8; Dojindo, Kumamoto, Japan) assay was used to evaluate the inhibition of cell growth in response to varying concentrations of 5-FU (0, 5, 10, 20, 40, 80, 120, 160, 200 μg/ml). Briefly, cells were seeded onto 96-well plates at a density of 5 × 10^3^ cells per well in 100 μl of culture medium and incubated at 37 °C with 5% CO_2_. After incubation for 24 h, varying concentrations of 5-FU diluted with the culture medium were added to each well and co-incubated for another 24 h. Then 10 μl of CCK-8 reagent was administered to each well for 2 h incubation at 37 °C. The optical density (OD) was detected by a microplate reader at 450 nm. Each experiment was repeated three times and each measurement was conducted three times.

### Colony formation assay

Cells were seeded onto 6-well plates at a density of 500 cells per well for adhesion-dependent colony formation. 5-FU was added to the culture medium at a final concentration of 15 μg/ml and the culture medium that contained 5-FU was changed every 3–4 days. After 2 weeks, visible colonies were fixed with 4% paraformaldehyde for 15 min and stained with 0.1% crystal violet staining solution for 10 min. Then, the formed colony units were photographed and counted for analysis.

### Enzyme-linked immunosorbent assay (ELISA)

The concentrations of CXCL5 and CCL18 in culture supernatants from M0, MS, MR, MKN45-S, HGC27-S, MKN45-R and HGC27-R cells (1 × 10^6^ cells) were quantified by ELISA kits (Cell Signaling Technology, MA, USA) according to the manufacturer’s instructions. Absorbance was measured using a microplate reader. The concentration of the sample were estimated from the standard curve and the levels below the detection limit of the assay were perceived as zero.

### Recombinant protein

Recombinant human CXCL5 (rhCXCL5) were purchased from R&D Systems (Minneapolis, MN, USA). 100 μg/ml stock solution of rhCXCL5 was achieved by dissolving 25 μg powder in 250 μl PBS, followed by adding 0.1% BSA in the final solution, and cells were treated with 10 ng/ml rhCXCL5 for 48 h.

### Chemotaxis assay

THP-1 cells were seeded onto the upper chamber (6-well plates, 0.8 μm pore size; Corning, NY, USA) at a density of 2 × 10^5^ in 200 μl serum-free medium. M0, MS and MR cells were cultured with serum-free medium for 24 h, then the supernatants from above cells were collected and added to the corresponding lower chamber with or without CXCL5 neutralizing antibody (0.5 μg/ml; Abcam, Cambridge, UK). After incubation for 24 h at 37 °C with 5% CO_2_, THP-1 cells that migrated to the lower chamber were measured by fixing and staining the inserts with 0.1% crystal violet staining solution and counting under a microscope (100-fold fields). Non-migratory cells were removed before the membrane was observed.

### RNA extraction and real-time quantitative polymerase chain reaction (RT-qPCR)

Total RNA was extracted from cells using TRIzol reagent (Invitrogen, Carlsbad, CA, USA) according to the manufacturer’s protocol. cDNA was synthesized from 1 μg of total RNA using 5 × PrimeScript RT reagent Kit (Takara, Dalian, China), and RT-qPCR was performed using TB Green Premix Ex Taq II (Takara, Dalian, China). Reactions were performed in triplicate and the relative mRNA expression was analyzed by the 2^−ΔΔCt^ method using GAPDH as an internal control. The forward and reverse primer sequences for the targeted genes are listed in Additional file 2: Table S1.

### Protein extraction and western blot analysis

Total protein was extracted out of cells using ice-cold RIPA buffer (Thermo Scientific, Rockford, IL, USA) with Halt Protease and Phosphatase inhibitor Cocktail (Thermo Scientific, Rockford, IL, USA) for 15 min. Protein samples were sonicated followed by centrifugation at 12000 g for 15 min at 4 °C and the concentrations were detected by BCA Protein Assay Kit (Beyotime, Shanghai, China). Approximately 30 μg of denatured protein was fractionated by 10% sodium dodecyl sulfate–polyacrylamide gel electrophoresis (SDS-PAGE) and transferred onto 0.45 μm polyvinylidene fluoride (PVDF) membranes (Millipore, Billerica, MA, USA). The PVDF membranes were blocked by TBST solution containing 5% skimmed milk for 1 h at room temperature and then incubated overnight at 4˚C with the primary antibodies against P-gp, Bcl-2, Bax, PTEN, PI3K, p-PI3K, AKT, p-AKT, mTOR, p-mTOR (1:1000, Cell Signaling Technology, MA, USA) and GAPDH (1:500, Cell Signaling Technology, MA, USA). Next, the membranes were incubated with corresponding secondary antibody HRP-conjugated Anti-Rabbit IgG (1:10000, Cell Signaling Technology, MA, USA) at room temperature for 1 h. SuperSignal West Pico PLUS Chemiluminescent Substrate (Thermo Scientific, Rockford, IL, USA) was used for visualizing the blots in a Kodak Image station (Tanon, China) (Additional file 3: Table S2).

### Flow cytometry analysis

M0, MS and MR cells were washed twice by PBS and filtered through a 100 μm mesh for flow cytometry. Then cells were counted, diluted to 1 × 10^6^ cells per 100 μl and subsequently stained with FITC-CD11b, PE-CD86, APC-CD163 and APC-CD206 antibodies (BioLegend, San Diego, CA, USA) followed by incubating in darkness at 4 °C for 15 min. Finally, the labeled cells were analyzed by BD Accuri C6 Plus flow cytometer (BD Biosciences, San Jose, CA, USA). The process was conducted in triplicate and data were analyzed by FlowJo software (Tree Star, Oregon, OR, USA) (Additional file 4: Figure S1).

### Apoptosis assay

Cell apoptosis was measured using Annexin-V-FITC Apoptosis Detection Kit (Dojindo, Kumamoto, Japan) according to the manufacturer’s protocol. In brief, cells were washed twice with PBS, after centrifugation, cells were suspended in 100 μl of 1 × binding buffer. Then 5 μl Annexin V-FITC and 5 μl propidium iodide (PI) were added to stain cells for 15 min in the dark. The stained cells were maintained on ice until apoptosis was measured using BD Accuri C6 Plus flow cytometer (BD Biosciences, San Jose, CA, USA). The process was conducted in triplicate and data were analyzed by FlowJo software (Tree Star, Oregon, OR, USA).

### Statistical analysis

Data were expressed as mean ± standard deviation (SD) of at least three separate experiments. Student’s t-test or one-way analysis of variance (ANOVA) was used for difference analysis. Correlation of the expression level of CD163 or CD206 with CXCL5 was determined using Spearman rank-order correlation. Survival curves were analyzed using Kaplan–Meier and log-rank methods. All statistical analyses were calculated by SPSS 22.0 (SPSS Inc., Chicago, IL, USA) in conjunction with GraphPad Prism 8 (GraphPad Prism Software, Inc., San Diego, CA, USA), and P < 0.05 was considered statistically significant.

## Results

### Infiltration of macrophages was associated with the resistance of gastric cancer to chemotherapy

**Fig. 1:**
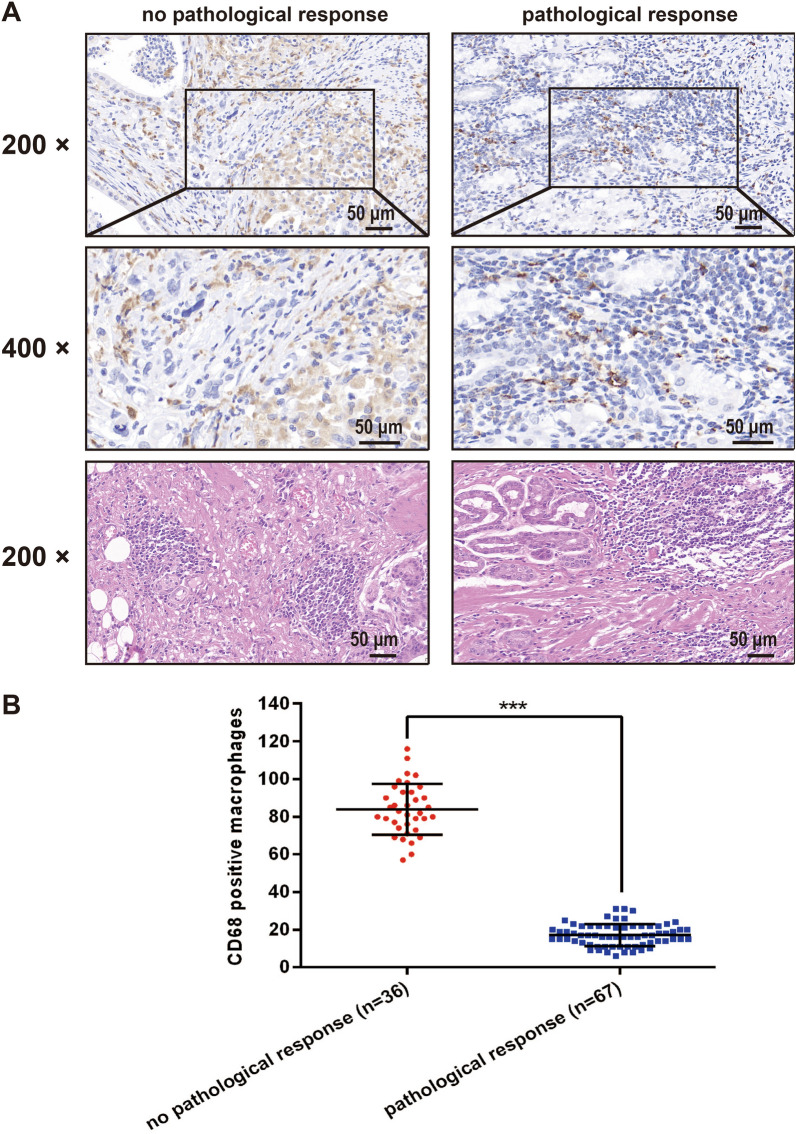
Infiltration of macrophages was associated with the resistance of gastric cancer to chemotherapy. **A** The macrophages in tumor tissue of gastric cancer were assessed by IHC staining. CD68 positive macrophages were counted in 5 random 400-fold fields. **B** The number of macrophages in no pathological response group (n = 36) was significantly increased compared with that of pathological response group (n = 67). Data were statistically analyzed with Student’s t-test, and values were presented as mean ± SD. ***P < 0.001

To investigate the biodistribution of macrophages in gastric cancer, we detected the expression of CD68, one of the markers of macrophages, in 103 clinical samples from patients receiving 5-FU based neoadjuvant chemotherapy by immunohistochemistry. CD68 positive macrophages were counted in 5 random 400-fold fields of each section. Results showed that the number of macrophages infiltrated in tissue from no pathological response group was significantly increased compared with that of pathological response group (Fig. 1, p < 0.001). The above finding indicated that infiltration of macrophages may play a critical role in the development and progression of chemoresistance in gastric cancer.

### Cells acquired chemoresistance via long-term 5-FU inducing

**Fig. 2:**
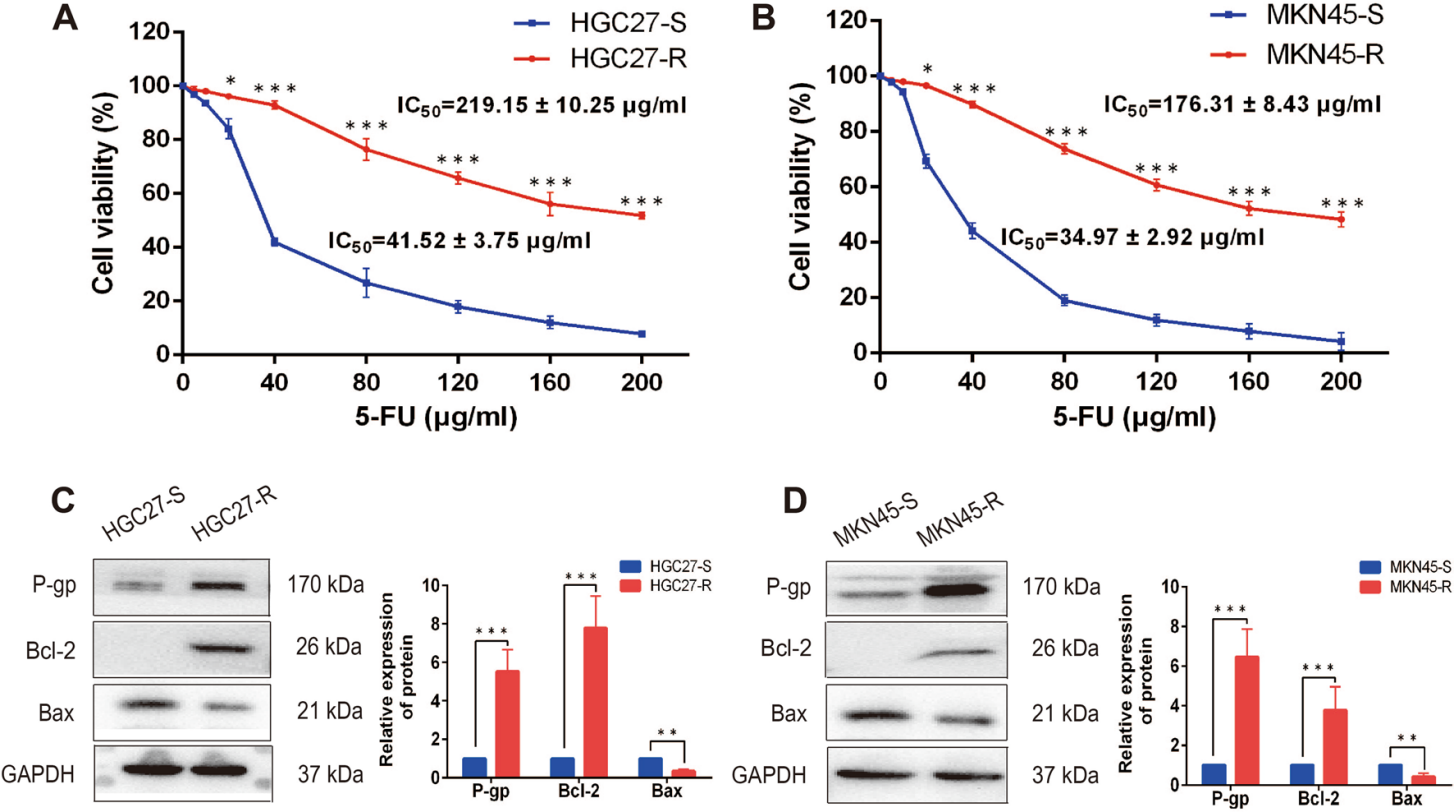
Cells acquired chemoresistance via long-term 5-FU inducing. **A** and **B** MKN45-R, HGC27-R cells and their parental cells MKN45-S and HGC27-S cells were cultured in the presence of 5-FU for 24 h, cell viability was detected by CCK-8 assay. **C** and **D** Western blot analysis indicated the expression of P-gp and Bcl-2 were significantly increased, and Bax was significantly decreased in MKN45-R and HGC27-R cells compared with parental cells. Data were presented as mean ± SD of three independent experiments. Student’s t-test was performed for comparisons. *P < 0.05, **P < 0.01 and ***P < 0.001. 5-FU, 5-fluorouracil

Two 5-FU-resistant gastric cell lines, MKN45-R and HGC27-R, were established by continuous exposure of the parental cells to increasing concentrations of 5-FU. To confirm that they were resistant to 5-FU, increasing concentrations of 5-FU (0, 5, 10, 20, 40, 80, 120, 160, 200 μg/ml) were added and cell viability was then detected by CCK-8 assay. The results demonstrated that 5-FU decreased cell viability in a dose-dependent manner (Fig. 2A, B). The IC_50_ values of MKN45-R and HGC27-R cells increased to 176.31 ± 8.43 μg/ml and 219.15 ± 10.25 μg/ml respectively, remarkably higher compared with their parental cells MKN45-S (34.97 ± 2.92 μg/ml) (p < 0.001) and HGC27-S (41.52 ± 3.75 μg/ml) (p < 0.001). In addition, western blot analysis indicated that the expression levels of chemoresistance-associated protein P-gp (HGC27: p < 0.001; MKN45: p < 0.001) and anti-apoptotic protein Bcl-2 (HGC27: p < 0.001; MKN45: p < 0.001) were significantly increased, and pro-apoptotic protein Bax (HGC27: p = 0.004; MKN45: p = 0.006) was significantly decreased in MKN45-R and HGC27-R cells (Fig. 2C, D). Uncropped western blot images were demonstrated in Additional file 5: Figure S2. As a result, the above data denoted that 5-FU-resistant gastric cell lines were effectively established.

### Co-culturing gastric cancer cells induced macrophages polarization to M2 phenotype

**Fig. 3:**
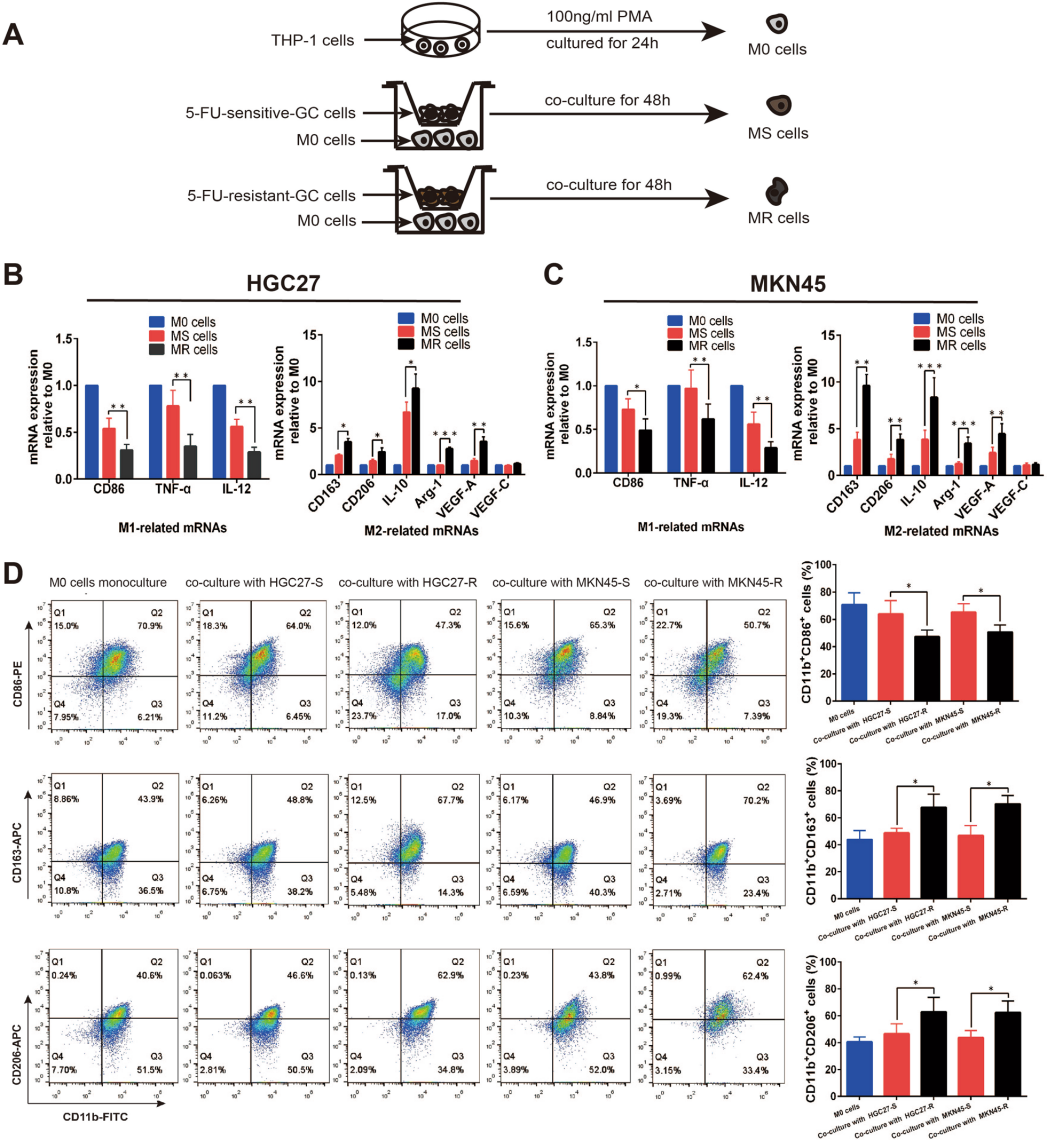
Co-culturing gastric cancer cells induced macrophages polarization to M2 phenotype. **A** Procedure used to induce and obtain M0 macrophages from THP-1 cells, MS macrophages from 5-FU-sensitive TME and MR macrophages from 5-FU-resistant TME. **B** and **C** The levels of M1-related gene expression of CD86, TNF-α and IL-12, and M2-related genes expression of CD163, CD206, IL-10, Arg-1, VEGF-A and VEGF-C in M0, MS and MR cells were analyzed by RT-qPCR. **D** The percentages of CD86^+^CD11b^+^ cells (M1 macrophages), CD163^+^CD11b^+^ cells (M2 macrophages), and CD206^+^CD11b^+^ cells (M2 macrophages) were measured through flow cytometry analysis. Data were presented as mean ± SD of three independent experiments. Student’s t-test was performed for comparisons. *P < 0.05, **P < 0.01 and ***P < 0.001. PMA, phorbol 12-myristate 13-acetate; 5-FU, 5-fluorouracil; GC, gastric cancer

Human monocyte cell line THP-1 was differentiated into macrophage (M0) via treatment with 100 ng/ml PMA for 24 h. M0 macrophages were subsequently co-cultured with MKN45-R, HGC27-R cells and their parental cells respectively in a non-contact Transwell system for 48 h, and then TAMs from 5-FU-sensitive TME (MS) and 5-FU-resistant TME (MR) were obtained (Fig. 3A). RT-qPCR was performed to explore the effect of co-culturing gastric cells on M0 cells. As a result, the levels of M1-related gene expression of CD86, TNF-α and IL-12 were down-regulated in MS and MR cells relative to M0 cells. Furthermore, the expression levels were significantly lower in MR cells than in MS cells (Fig. 3B, C). In contrast, the levels of M2-related genes expression of CD163, CD206, IL-10, Arg-1 and VEGF-A were up-regulated after co-culturing with gastric cells, and the expression levels were remarkably higher in MR cells than in MS cells (Fig. 3B, C). Flow cytometry analysis was used to characterize the surface makers of M0, MS and MR cells. It showed that the percentage of CD86^+^CD11b^+^ cells (M1 macrophages) was decreased in MS and MR cells, and the percentage was significantly lower in MR cells than in MS cells (HGC27: p = 0.032; MKN45: p = 0.028). Besides, the percentages of CD163^+^CD11b^+^ (HGC27: p = 0.040; MKN45: p = 0.029) and CD206^+^CD11b^+^ cells (HGC27: p = 0.016; MKN45: p = 0.039) (M2 macrophages) were increased in MS and MR cells, and the percentage was higher in MR cells than in MS cells (Fig. 3D). Taken together, the above results indicated that gastric cancer cells can modulate macrophages to M2-like polarization, and chemoresistant cells seem to be more effective in inducing macrophages polarization to M2 phenotype.

### Co-culturing M2-polarized macrophages enhanced 5-FU-resistance in gastric cancer cells

**Fig. 4:**
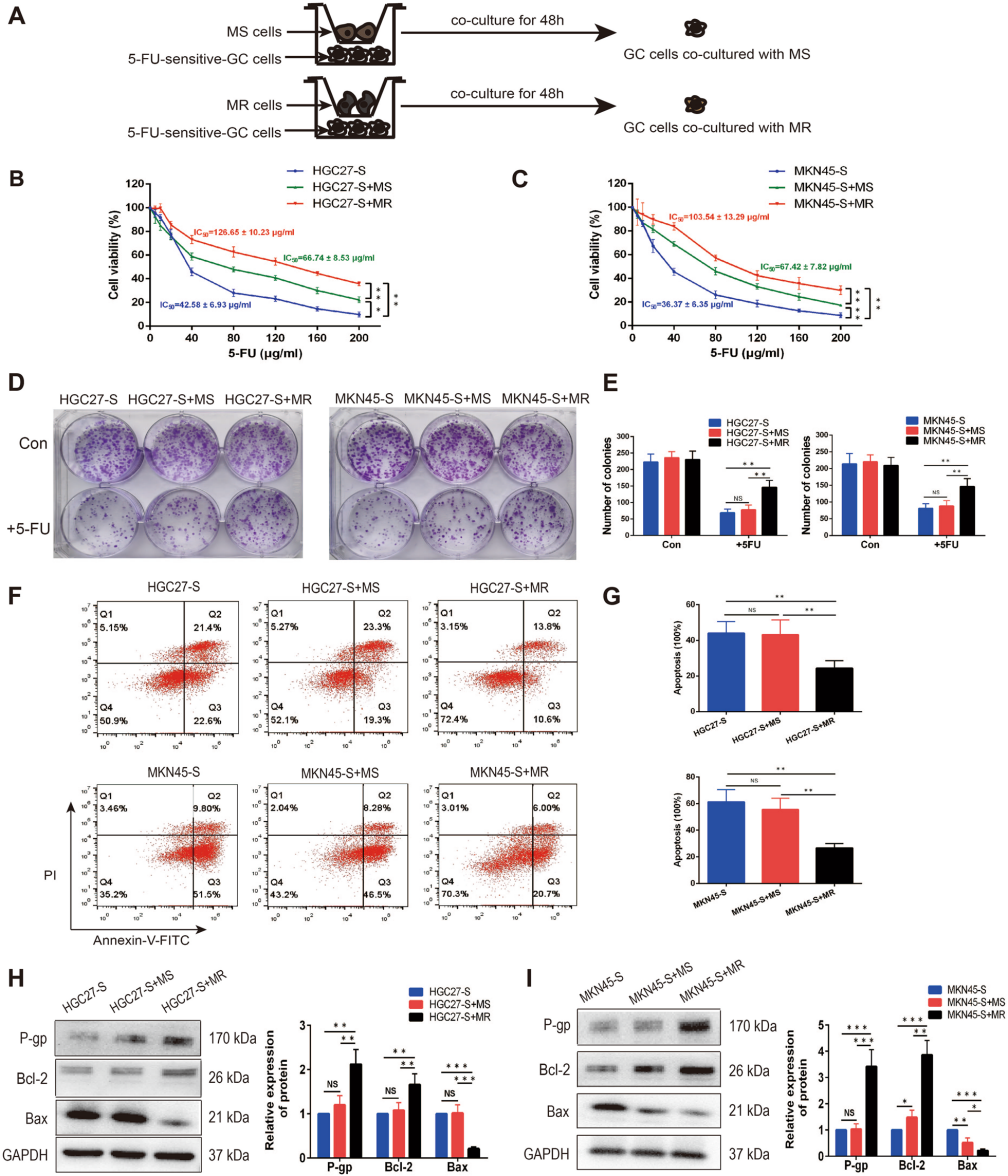
Co-culturing M2-polarized macrophages enhanced 5-FU-resistance in gastric cancer cells. **A** Procedure used to illustrate the effect of MS and MR macrophages on gastric cancer cells. **B** and **C** CCK-8 assay indicated that culturing with MS and MR macrophages induced 5-FU-resistance and enhanced the IC_50_ values of HGC27-S and MKN45-S cells. **D**–**G** Gastric cancer cells were cultured alone, co-cultured with MS and MR macrophages followed by being treated with or without 15 μg/ml 5-FU. The numbers of colonies were counted and the apoptosis was analyzed by apoptosis flow cytometry assay. **H** and **I** Western blot analysis indicated that the expression of P-gp and Bcl-2 were significantly increased, and Bax was significantly decreased in gastric cancer cells co-cultured with MR macrophages. Data were presented as mean ± SD of three independent experiments. Student’s t-test was performed for comparisons. *P < 0.05, **P < 0.01 and ***P < 0.001. NS, not significant (P > 0.05). 5-FU, 5-fluorouracil; GC, gastric cancer

Gastric cancer cells were co-cultured with macrophages of different phenotypes in a non-contact Transwell system for 48 h, then macrophages were discarded and gastric cancer cells were collected for further analysis (Fig. 4A). CCK-8 assay was adopted to investigate whether the resistance to 5-FU was affected by macrophages. As indicated in Fig. 4B and C, co-culturing with MS and MR macrophages enhanced the IC_50_ values of HGC27-S cells from 42.58 ± 6.93 μg/ml to 66.74 ± 8.53 and 126.65 ± 10.23 μg/ml respectively (p = 0.003; p = 0.006). Similarly, co-culturing with MS and MR macrophages enhanced the IC_50_ values of MKN45-S cells from 36.37 ± 6.35 μg/ml to 67.42 ± 7.82 and 103.54 ± 13.29 μg/ml respectively (p = 0.006; p = 0.002). In addition, cell colony formation assay also demonstrated that co-culturing MR macrophages significantly promoted 5-FU-resistance of gastric cancer cells, whereas the effect of MS macrophages was remarkably smaller than that of MR macrophages (Fig. 4D, E; HGC27: p = 0.003; MKN45: p = 0.005). Chemotherapy-induced apoptosis was also determined through apoptotic flow cytometry assay. In brief, after co-culturing with MS and MR macrophages, gastric cancer cells were then treated with 5-FU at the concentration of 15 μg/ml for 24 h. The result indicated that the rate of apoptosis was significantly lower in gastric cancer cells co-cultured with MR macrophages as compared to that cultured alone or co-cultured with MS macrophages (Fig. 4F, G; HGC27: p = 0.002, p = 0.004; MKN45: p = 0.002, p = 0.003). Western blot analysis further indicated that the expression levels of chemoresistance-associated protein P-gp and anti-apoptotic protein Bcl-2 were significantly increased, and pro-apoptotic protein Bax was significantly decreased in gastric cancer cells co-cultured with MR macrophages (Fig. 4H, I; HGC27: p = 0.007, p = 0.005, p < 0.001; MKN45: p < 0.001, p < 0.001, p < 0.001). Uncropped western blot images were demonstrated in Additional file 6: Figure S3. All the findings above indicated that 5-FU-resistant gastric cancer cells can effectively induce macrophages polarization to M2 phenotype, which in turn promotes 5-FU-resistance in gastric cancer cells.

### CXCL5 derived from M2-polarized macrophages was associated with 5-FU-resistance of gastric cancer cells

**Fig. 5:**
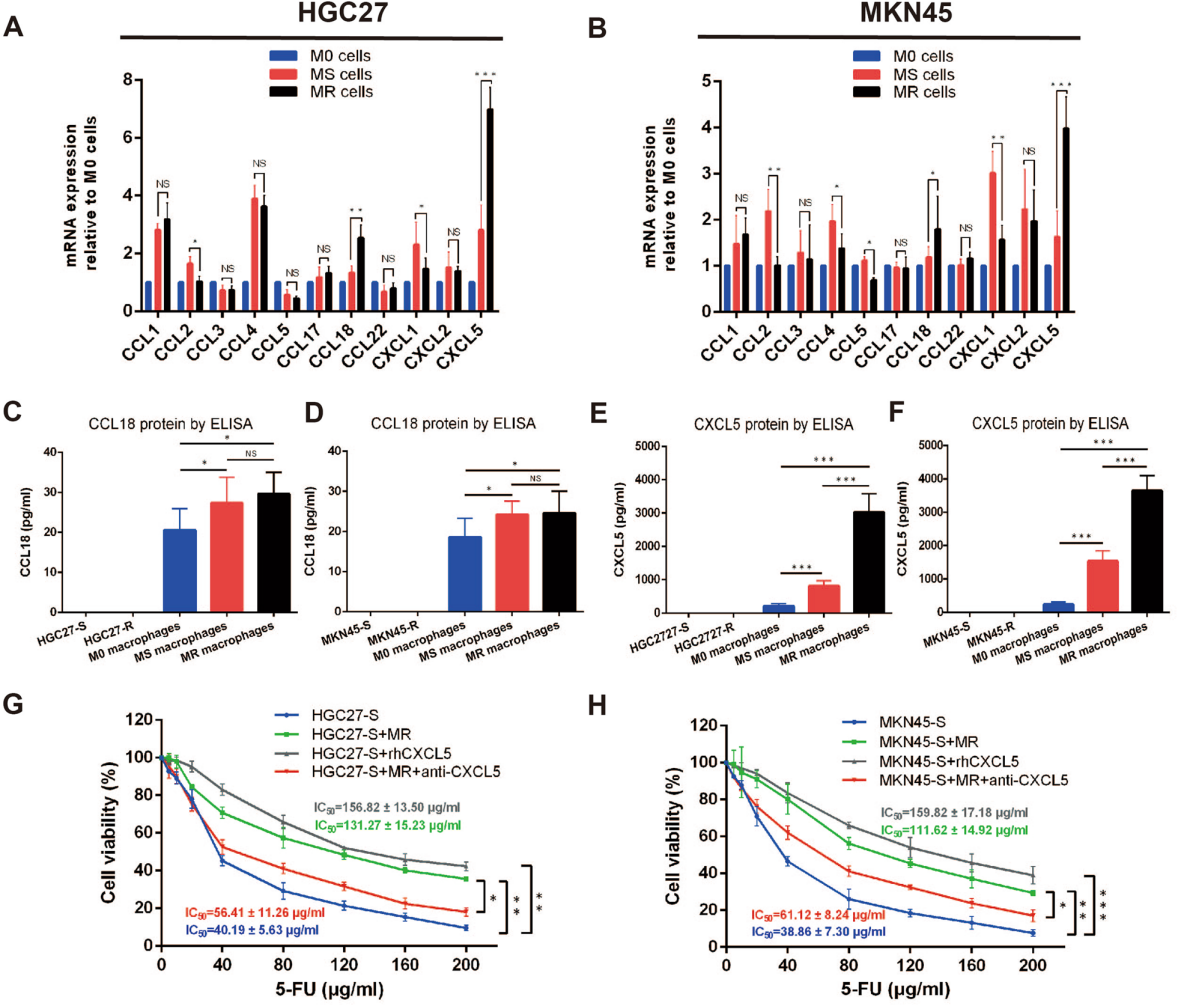
CXCL5 derived from M2-polarized macrophages was associated with 5-FU-resistance of gastric cancer cells. **A** and **B** RT-qPCR determined the mRNA levels of eleven cytokines in M0, MS and MR macrophages, the change trend of CCL18 and CXCL5 were consistent with previous results and the difference was statistically significant. **C**–**F** ELISA detected the levels of CCL18 and CXCL5 protein in culture supernatants. CCL18 and CXCL5 were mainly derived from macrophages rather than gastric cancer cells, by contrast to CCL18, CXCL5 levels in MS and MR macrophages were remarkably high and the difference was statistically significant. **G** and **H** CCK-8 assay was performed to verify the role of CXCL5 in 5-FU-resistance of gastric cancer cells. Data were presented as mean ± SD of three independent experiments. Student’s t-test was performed for comparisons. *P < 0.05, **P < 0.01 and ***P < 0.001. NS, not significant (P > 0.05). 5-FU, 5-fluorouracil; CCL, CC-chemokine ligand; CXCL, CXC motif chemokine ligand

It was speculated that the different ability of MS and MR macrophages to enhanced 5-FU-resistance of gastric cancer cells was due to the different level of secreted cytokines. After reviewing the relevant literature, eleven cytokines (CCL1, CCL2, CCL3, CCL4, CCL5, CCL17, CCL18, CCL22, CXCL1, CXCL2 and CXCL5) were selected for analyzing through RT-qPCR. As indicated in Fig. 5A and B, of the eleven cytokines, only CCL18 and CXCL5 whose transcription levels were consistent with the trend of previous results (Fig. 4B–I) and the transcription level in MR macrophages was significantly higher than that in MS macrophages. The levels of CCL18 and CXCL5 protein in culture supernatants were then further detected by ELISA. The results denoted that the levels of CCL18 and CXCL5 in 5-FU-sensitive and 5-FU-resistant gastric cancer cells were below the detection limit of the assay and recorded as zero (Fig. 5C-F). In addition, the concentrations of CCL18 in culture supernatants from MS and MR macrophages were very low and the difference was not statistically significant (Fig. 5C, D; HGC27: p = 0.149; MKN45: p = 0.372), which could not explain the difference between MS and MR macrophages in enhancing 5-FU-resistnace of gastric cancer cells. By contrast to CCL18, CXCL5 levels in MS and MR macrophages were remarkably high and the difference was statistically significant (Fig. 5E, F; HGC27: p < 0.001; MKN45: p < 0.001)**.** Based on the results, we focused on CXCL5 in the further studies to verify its role in 5-FU-resistance of gastric cancer cells. 5-FU-sensitive gastric cancer cells were cultured alone or co-cultured with MR macrophages with the presence of rhCXCL5 (10 ng/ml) or CXCL5 neutralizing antibody (0.5 μg/ml) for 48 h followed by being treated with different concentrations of 5-FU (0, 5, 10, 20, 40, 80, 120, 160, 200 μg/ml) for 24 h, then the cell survival rates were assessed. As indicated by CCK-8 assay, co-culturing MR macrophages or addition of rhCXCL5 could induce 5-FU-resistance, whereas CXCL5 neutralizing antibody decreased MR macrophages-mediated resistance to 5-FU in gastric cancer cells (Fig. 5G, H). Taken together, the above results denoted that CXCL5 derived from M2-polarized macrophages was one of the major chemokines that associated with 5-FU-resistance in gastric cancer cells.

### M2-polarized macrophages inhibited apoptosis and increased 5-FU-resistance by activing the CXCL5/PI3K/AKT/mTOR pathway in gastric cancer cells.

**Fig. 6:**
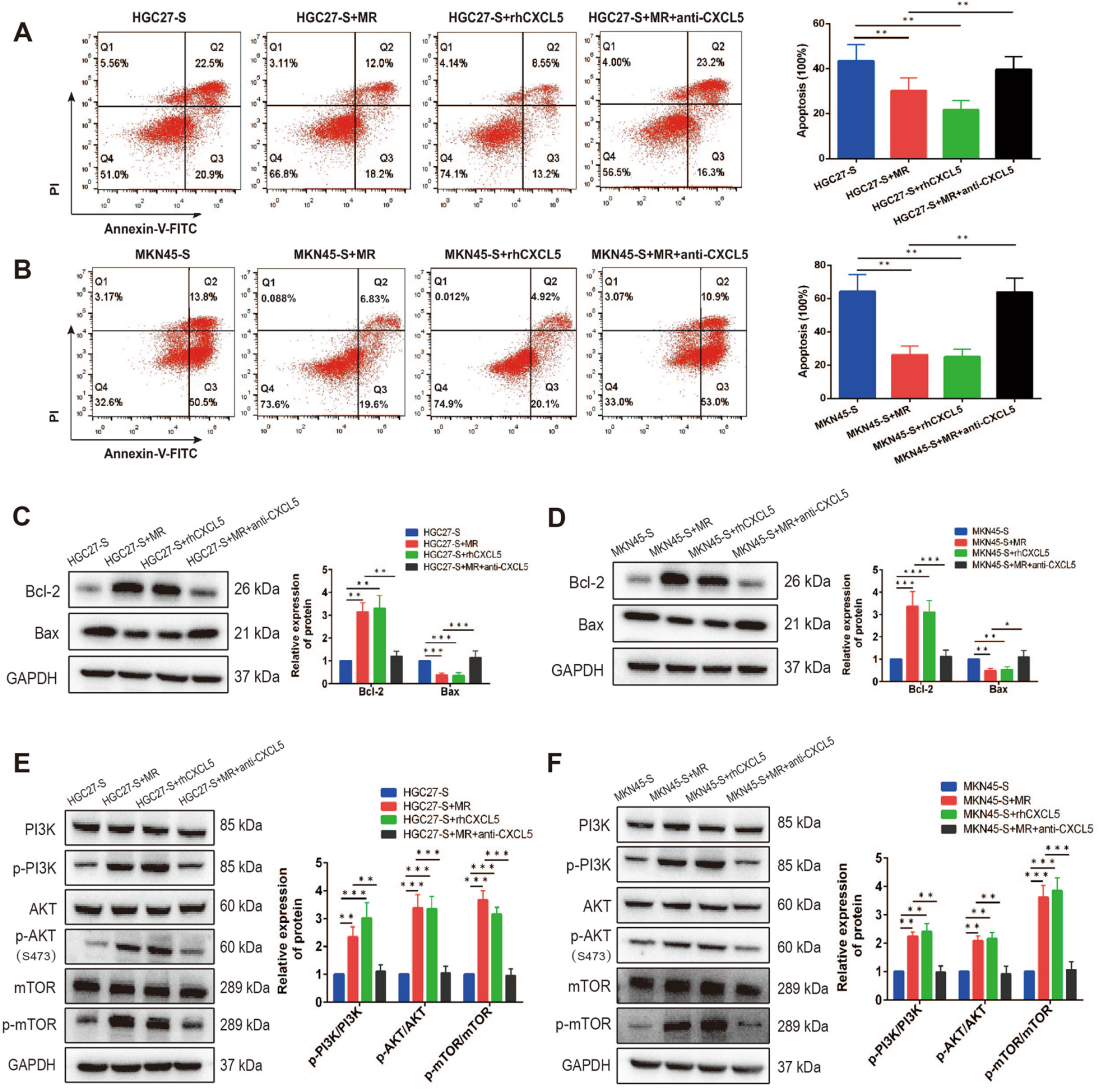
M2-polarized macrophages inhibited apoptosis and increased 5-FU-resistance by activing the CXCL5/PI3K/AKT/mTOR pathway in gastric cancer cells. **A** and **B** Apoptotic flow cytometry assay was operated to verify that MR macrophages protected gastric cancer cells from 5-FU-induced apoptosis via CXCL5. **C** and **D** Western blot analysis indicated that MR macrophages regulated the expression levels of anti-apoptotic protein Bcl-2 and pro-apoptotic protein Bax. **E** and **F** Western blot of PI3K/AKT/mTOR pathway proteins were analyzed. Co-culturing MR macrophages and rhCXCL5 could activate the PI3K/AKT/mTOR pathway in gastric cancer cells, CXCL5 neutralizing antibody remarkably weakened the ability of co-culturing MR macrophages to activate this signaling pathway. Data were presented as mean ± SD of three independent experiments. Student’s t-test was performed for comparisons. *P < 0.05, **P < 0.01, ***P < 0.001

There is accumulating evidence indicating that resistance to apoptosis is responsible for chemoresistance. 5-FU exerts the anti-tumor efficiency mainly though mediating apoptosis of tumor cells. Therefore, we determined whether M2-polarized macrophages mediated 5-FU-resistance through regulation of apoptosis in gastric cancer cells. 5-FU-sensitive gastric cancer cells were cultured alone or co-cultured with MR macrophages with the presence of rhCXCL5 (10 ng/ml) or CXCL5 neutralizing antibody (0.5 μg/ml) for 48 h followed by being treated with 15 μg/ml 5-FU for 24 h, then the rates of apoptosis were analyzed. It was revealed that co-culturing MR macrophages or rhCXCL5 could both reduce apoptotic proportion, however, applying CXCL5 neutralizing antibody decreased MR macrophages-mediated resistance to apoptosis in gastric cancer cells (Fig. 6A, B). Western blot analysis further denoted that co-culturing MR macrophages or addition of rhCXCL5 increased the expression levels of anti-apoptotic protein Bcl-2, whereas pro-apoptotic protein Bax was significantly decreased in gastric cancer cells. In addition, the ability of MR macrophages to affect the expression of Bcl-2 and Bax was remarkably weakened by CXCL5 neutralizing antibody (Fig. 6C, D). Previous studies have revealed that the PI3K/AKT/mTOR signaling pathway plays a critical role in tumor progression through regulating cell proliferation, apoptosis and chemoresistance. Based on this, the potential effect of M2-polarized macrophages on the activation of the PI3K/AKT/mTOR pathway was further investigated. As indicated in Fig. 6E and F, co-culturing MR macrophages and rhCXCL5 could both significantly increased the expression of phosphorylated PI3K, AKT and mTOR, whereas treatment with CXCL5 neutralizing antibody remarkably weakened the ability of co-culturing MR macrophages to activate this signaling pathway. Uncropped western blot images were demonstrated in Additional file 7: Figure S4. Taken together, the above results indicated that CXCL5 derived from M2-polarized macrophages inhibited apoptosis and activated the PI3K/AKT/mTOR pathway to increase 5-FU-resistance in gastric cancer cells.

### CXCL5 recruited monocytes to promote the development of chemoresistant microenvironment in gastric cancer

**Fig. 7:**
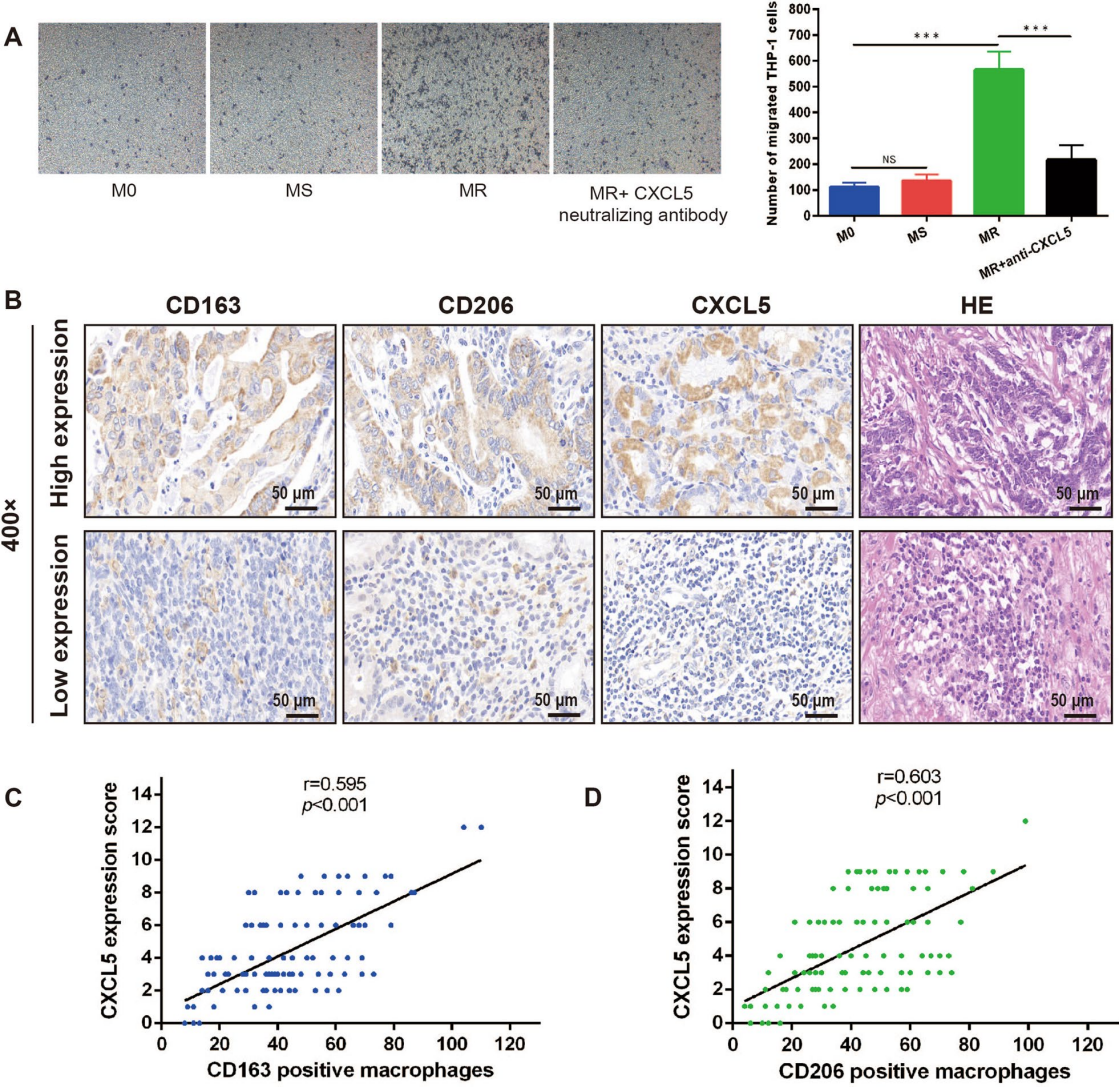
CXCL5 recruits monocytes to promote the development of chemoresistant microenvironment in gastric cancer. **A** Representative photos of THP-1 cells recruited by different conditioned medium with or without CXCL5 neutralizing antibody (100 ×), the quantitative results verified that CXCL5 could recruit monocytes. Data were presented as mean ± SD of three independent experiments. Student’s t-test was performed for comparisons. **B** Representative images of immunohistochemical staining for CD163, CD206, CXCL5 and HE staining in human gastric cancer tissues. (C and D) CXCL5’s association with CD163 and CD206 in gastric cancer tissue was analyzed through Spearman rank-order correlation (n = 103). ***P < 0.001. NS, not significant (P > 0.05). CXCL, CXC motif chemokine ligand

To identify how macrophages affected the recruitment of monocytes in gastric cancer, chemotaxis assay was performed. It has already been determined that the levels of CXCL5 were different in M0, MS and MR macrophages, we speculated that the effect of macrophages on recruitment of monocytes coincided with the secretion level of CXCL5. Data from chemotaxis assay indicated that M0, MS and MR macrophages could attract THP-1 cells, the chemotaxis-inducing effect of MR macrophages was significantly stronger than that of M0 and MS macrophages (p < 0.001; p < 0.001), and the CXCL5 neutralizing antibody could weakened the MR macrophages-induced chemotaxis in gastric cancer (Fig. 7A; p < 0.001). To further verified the role of M2-polarized macrophages-derived CXCL5 in gastric cancer, the expressions of CXCL5, CD163 and CD206 in clinical samples from 103 patients were determined by immunohistochemistry. The result demonstrated that the high expression of CXCL5 correlated with the high infiltration of CD163 positive macrophages (r = 0.595, p < 0.001) and CD206 positive macrophages (r = 0.603, p < 0.001) (Fig. 7B-D). Based on the data above, we speculated that gastric cancer cells, especially chemoresistant cells could induce macrophages polarization to M2 phenotype, which lead to the increased secretion of CXCL5. High level of CXCL5 could in turn recruit monocytes to form more M2-polarized macrophages and further promote the development of chemoresistant microenvironment in gastric cancer.

### High expression of CXCL5 in gastric cancer tissue correlated with poor prognosis of patients

**Fig. 8:**
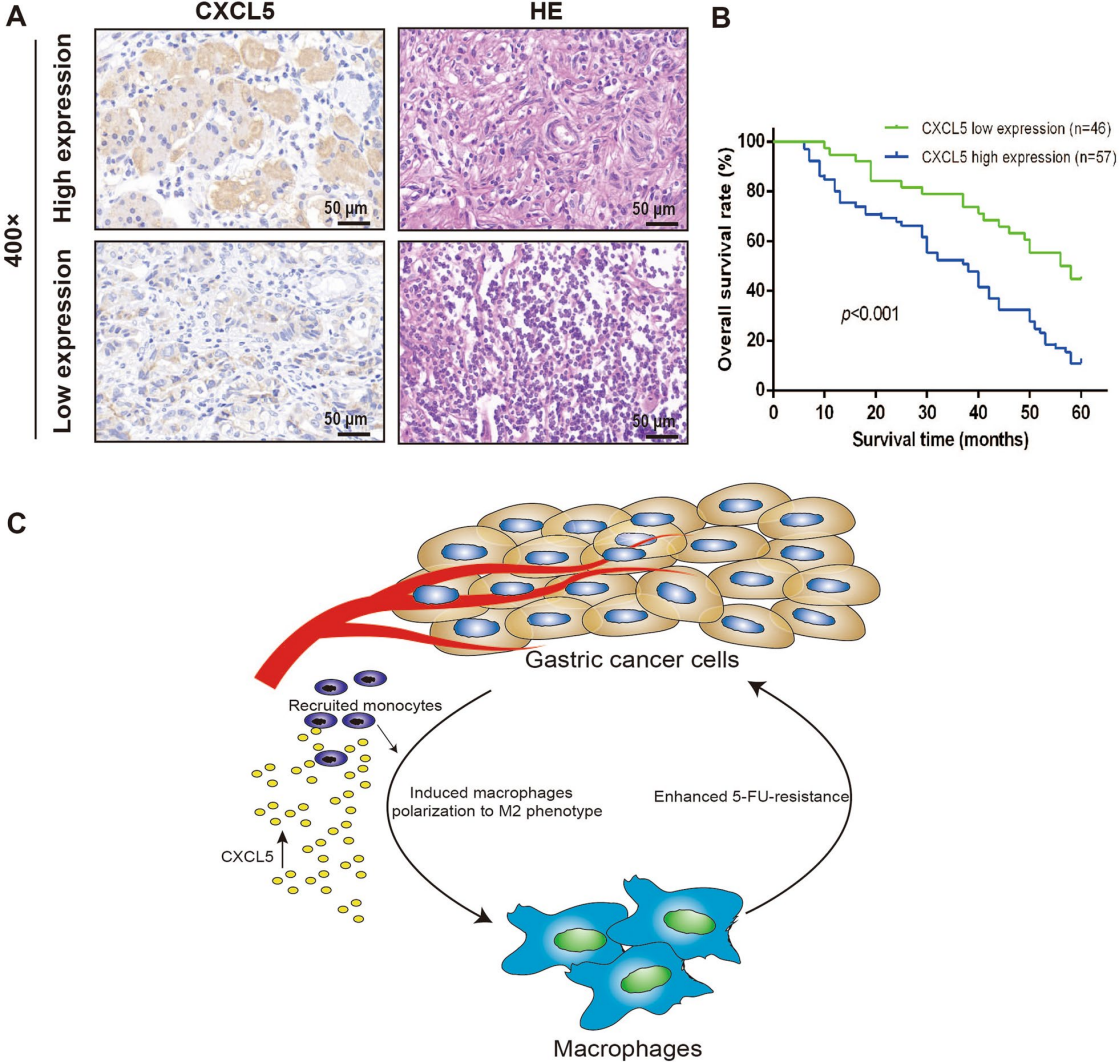
High expression of CXCL5 in gastric cancer tissue correlated with poor prognosis of patients. **A** Expression of CXCL5 in gastric cancer tissue was analyzed by immunohistochemistry, and different patterns of CXCL5 immunohistochemical staining were shown. **B** Kaplan–Meier analysis and the log-rank test were used to evaluate the correlation between CXCL5 expression and overall survival in patients with gastric cancer (n = 103). **C** Graphical abstract to show how macrophages interacted with gastric cancer cells to promote the development of chemoresistant microenvironment in gastric cancer. CXCL, CXC motif chemokine ligand; 5-FU, 5-fluorouracil

To investigate the clinical relevance of CXCL5 in prognosis of patients with gastric cancer, we detected the expression level of CXCL5 in clinical samples from 103 patients by immunohistochemistry. The representative images of low (n = 46) and high (n = 57) expression of CXCL5 were showed in Fig. 8A**.** Furthermore, the survival analysis indicated that the overall survival (OS) rates of patients with higher expression of CXCL5 were significantly lower than that of patients with lower expression of CXCL5 (Fig. 8B; p < 0.001). Taken together, the above results demonstrated that CXCL5 can be used as an effective biomarker for predicting chemoresistant and prognosis in gastric cancer.

## Discussion

Perioperative chemotherapy combing with surgical operation is currently the main treatment for patients with advanced gastric cancer, and 5-FU-based chemotherapy is now the most widely used chemotherapeutic criterion in clinical practice. However, chemoresistance is one of the major obstacles to achieving effective chemotherapy, resulting in chemotherapy failure and tumor progression [21]. In addition to the genetic variation of tumor cells themselves causing enhanced anti-apoptotic ability and increased drug efflux, it has been increasingly approved that chemoresistance is a complex process of dynamic interactions between TME and tumor cells [6, 22, 23]. An increasing body of evidence demonstrated that TAMs are among the most important regulator in the TME and TAMs-related therapies have been considered prospective strategies for malignant tumors, including gastric cancer [18, 24–26]. Clinical studies have showed the correlation between the high infiltration of TAMs and the poor prognosis in several types of malignant tumors [14, 15, 27, 28]. However, whether TAMs involved the development of chemoresistance in gastric cancer has not been elucidated so far. The present study demonstrated the interaction between TAMs and the chemoresistant phenotype of tumor cells in gastric cancer for the first time.

As the phenotypes of TAMs are heterogeneous and plastic [11], we detected the total macrophages infiltration rather than M1 or M2 macrophages in gastric cancer tissues to explore the clinical value of TAMs in the development of resistance to chemotherapy. Interestingly, the results of staining data from 103 patients demonstrated that the high infiltration of TAMs was significantly associated with the chemoresistance of gastric cancer. To mimic the in vivo tumor microenvironment and explore the interaction between TAMs and gastric cancer cells, we firstly generated 5-FU-resistant gastric cell lines by exposing cells to increasing concentrations of 5-FU, and then the indirect cell–cell interactions were measured after co-culturing TAMs with gastric cancer cells. We observed that 5-FU-resistant gastric cancer cells were more effective than 5-FU-sensitive gastric cancer cells in skewing macrophages to M2 polarization, characterized by up-regulated expression of CD163, CD206, IL-10, Arg-1 and VEGF-A and down-regulated expression of CD86, TNF-α and IL-12. Consistent with the changes in the expression of P-gp and apoptosis-related proteins, M2-polarized TAMs, in turn, enhanced the ability of 5-FU-resistance and anti-apoptosis of gastric cancer cells via indirect co-culture, suggesting that some soluble factors secreted from TAMs affect the chemoresistance of gastric cancer cells. Many macrophages-derived cytokines present in the TME have already been verified to affect the response of cancer cells to chemotherapy like CCL2 [16], CCL22 [17], IL-6 [19] and CCL18 [29]. Given the critical role of cytokines in cell–cell interaction, we applied RT-qPCR-based cytokines array analysis, combining with ELISA, to screen the changes of transcription level of cytokines in three macrophages (M0, MS and MR) and identified CXCL5 as the rational target that accountable for TAMs-induced chemoresistance in gastric cancer.

As a member of the Glu-Leu-Arg (ELR) positive CXC chemokine family, CXCL5 has been identified as an inflammatory mediator with critical role in malignant tumors [30, 31]. Many studies have demonstrated that CXCL5 could promote cancer progression via the receptor CXCR2 [32, 33]. CXCL5-mediated ERK/Snail signaling increased the potential of metastases in breast cancer [34]. In nasopharyngeal carcinoma, CXCL5/CXCR2 axis promoted cell migration and invasion by inducing EMT through ERK/GSK-3β/Snail signalling pathway [35]. In addition, CXCL5 could promote migration of gastric cancer cells via activating CXCR2/STAT3 feed-forward loop, CXCR2 was found to overexpress in gastric cancer tissue and the expression of CXCR2 was higher in six different gastric cancer cell lines, including the two gastric cancer cell lines used in our study, compared to that in a normal gastric epithelium cell line [36]. In the present study, TAMs-derived CXCL5 was demonstrated to promote 5-FU-resistance and enhance the ability of anti-apoptosis of gastric cancer cells through the activation of PI3K/AKT/mTOR pathway, which has been shown to involve in cancer progression. Emerging evidence demonstrated that the aberrant activation of PI3K/AKT/mTOR pathway could modulate epithelial-mesenchymal transition, autophagy, chemoresistance and metastasis in several human cancers [17, 37–39]. Also, PI3K/AKT/mTOR pathway has been shown to play a significant role in the promotion of cell survival through the modulation of apoptosis-related genes such as Bcl-2 and Bax [40]. Consistent with these researches, we demonstrated that the co-culturing with M2-polarized macrophages or the addition of rhCXCL5 could activate PI3K/AKT/mTOR pathway, marked by the increasing expression of phosphorylated PI3K, AKT and mTOR, whereas the treatment of CXCL5 neutralizing antibody remarkably reversed this effect.

As for THP-1 cells, they have also been identified to express receptor for CXCL5, namely CXCR2 [41, 42]. Consistently, our study showed that CXCL5 induced the aggregation of monocytes into the TME, and the transition from monocytes to TAMs further promoted the development of chemoresistant microenvironment. Moreover, the staining data confirmed the correlation between the expression of CXCL5 and the density of M2-polarized macrophages, and patients with high expression of CXCL5 in gastric cancer lesions had low overall survival rates.

Some limitations of the present study should be mentioned. First, THP-1 cells were pretreated with PMA to obtain differentiated macrophage-like cells at present study, whereas the primary macrophages from gastric cancer patients would make the study more persuasive. Interaction between tumor cells and TAMs is complicated and involved cytokines, metabolites and exosomes [43, 44]. In addition to TAMs, fibroblasts, lymphocytes, adipose cells and dendritic cells are included in the cell components of TME, and have been demonstrated to promote tumor malignant progression [45–48]. In our study, we only focused on the chemokines CXCL5 derived from TAMs and illuminated its critical role in 5-FU-resistnace, without evaluating its role in the invasion, angiogenesis or metastasis of gastric cancer, future study should focus on the effect of other components of the TME on the malignant progression in gastric cancer. Furthermore, the exact mechanism of how gastric cancer cells induced macrophages polarization to M2 phenotype should be further explored.

## Conclusion

In summary, we demonstrated that chemoresistant gastric cancer cells were more effective in inducing macrophages polarization to M2 phenotype, which in turn further promoted the chemoresistance of gastric cancer cells, thus forming a positive feedback loop between TAMs and gastric cancer cells (Fig. 8C). TAMs-derived CXCL5 played a critical role in the cell–cell interaction. CXCL5 could recruit monocytes to form more M2-polarized macrophages and further promote the development of chemoresistance through the activation of PI3K/AKT/mTOR pathway in gastric cancer. These findings suggested that targeting TAMs and blocking the cell–cell crosstalk between TAMs and gastric cancer cells may represent prospective therapeutic strategies for patients with chemoresistant gastric cancer.

## Supplementary Information

**Additional file 1: Text. S1.** Method of detaching PMA-treated THP-1 cells from the culture dish.

**Additional file 2: Table S1. **Primers used for real-time quantitative reverse transcription polymerase chain reaction.

**Additional file 3: Table S2. **Antibody used in the study.

**Additional file 4:** **Figure S1.** Gating strategy for flow cytometry.

**Additional file 5:** **Figure S2.** Uncropped western blot images of Fig. 2.

**Additional file 6:** **Figure S3.** Uncropped western blot images of Fig. 4.

**Additional file 7:** **Figure S4.** Uncropped western blot images of Fig. 6.

## Data Availability

The datasets used and/or analyzed during the current study are available from the corresponding author on reasonable request.

## Abbreviations

5-FU: 5-Fluorouracil
TME: Tumor microenvironment
TAMs: Tumor-associated macrophages
IHC: Immunohistochemistry
CXCL: CXC motif chemokine ligand
OS: Overall survival
IL: Interleukin
TNF: Tumor necrosis factor
VEGF: Vascular endothelial growth factor
Arg: Arginase
CCL: CC-chemokine ligand
CAP: College of American Pathologists
