# Supplementary material for: Crosstalk between tumor-associated macrophages and tumor cells promotes chemoresistance via CXCL5/PI3K/AKT/mTOR pathway in gastric cancer
Source: Cancer Cell Int. 2022 Sep 23;22:290. doi: 10.1186/s12935-022-02717-5 (PMC9508748; doi:10.1186/s12935-022-02717-5)
Supplement: Supplementary file 1 — Additional file 1: Text. S1. Method of detaching PMA-treated THP-1 cells from the culture dish. [file 12935_2022_2717_MOESM1_ESM.docx]

**Supplementary Text. S1**

Our method of detaching PMA-treated THP-1 cells from the culture dish was performed as follows: (1) Before we detached THP-1-derived macrophages, we put the cell culture dish on ice for 5-10 minutes to induce cell contraction and reduce the ability of adhesion. (2) 0.25% typsin containing 0.02% EDTA was placed in 37˚C incubator for 5 minutes to increase the enzymic activities. (3) Discard the culture medium followed by washing cell culture dish three times with PBS. (4) Add the warm trypsin for digestion (1ml per culture dish). Tap the bottom of the culture dish every two minutes during this period. (5) Approximately 15 minutes later, almost all cells can be detached from the culture dish, then culture medium was added to the culture dish to terminate the digestion. (6) Cells were collected into centrifuge tubes for centrifugation and then transferred to the chambers as needed.
