# Supplementary material for: Crosstalk between tumor-associated macrophages and tumor cells promotes chemoresistance via CXCL5/PI3K/AKT/mTOR pathway in gastric cancer
Source: Cancer Cell Int. 2022 Sep 23;22:290. doi: 10.1186/s12935-022-02717-5 (PMC9508748; doi:10.1186/s12935-022-02717-5)
Supplement: Supplementary file 2 — Additional file 2: Table S1. Primers used for real-time quantitative reverse transcription polymerase chain reaction. [file 12935_2022_2717_MOESM2_ESM.docx]

Supplementary Table S1. Primers used for real-time quantitative reverse transcription polymerase chain reaction

| Primer | |
| --- | --- |
| CD86  TNF-α  IL-12  CD163  CD206  IL-10  Arg-1  VEGF-A  VEGF-C  CCL1  CCL2  CCL3  CCL4  CCL5  CCL17  CCL18  CCL22  CXCL1  CXCL2  CXCL5 | 5’-TGGTGCTGCTCCTCTGAAGATTC-3’ (forward primer)  5’-ATCATTCCTGTGGGCTTTTTGTG-3’ (reverse primer)  5’-TGGAGAAGGGTGACCGACTC-3’ (forward primer)  5’-TGCCCAGACTCGGCAAAG-3’ (reverse primer)  5’-CGGTCATCTGCCGCAA-3’ (forward primer)  5’-AACCTAACTGCAGGGCACAG-3’ (reverse primer)  5’-CAGCGGCTTGCAGTTTCCTC-3’ (forward primer)  5’-TGAAATCAGCTGACTCATGGGAA-3’ (reverse primer)  5’-GGGAAAGGTTACCCTGGTGG-3’ (forward primer)  5’-GTCAAGGAAGGGTCGGATCG-3’ (reverse primer)  5’-GTCATCGATTTCTTCCCTGTG-3’ (forward primer)  5’-ACTCATGGCTTTGTAGATGCCT-3’ (reverse primer)  5’-TTCTCAAAGGGACAGCCACG-3’ (forward primer)  5’-TCAAGCAGACCAGCCTTTCT-3’ (reverse primer)  5’-CCTTGCTGCTCTACCTCCAC-3’ (forward primer)  5’-ATGATTCTGCCCTCCTCCTT-3’ (reverse primer)  5’-CACGAGCTACCTCAGCAAGA-3’ (forward primer)  5’-GCTGCCTGACACTGTGGTA-3’ (reverse primer)  5’-CTCATTTGCGGAGCAAGAGAT-3’ (forward primer)  5’-GCCTCTGAACCCATCCAACTG-3’ (reverse primer)  5’-CAGCCAGATGCAATCAATGCC-3’ (forward primer)  5’-TGGAATCCTGAACCCACTTCT-3’ (reverse primer)  5’-AGTTCTCTGCATCACTTGCTG-3’ (forward primer)  5’-CGGCTTCGCTTGGTTAGGAA-3’ (reverse primer)  5’-CTGTGCTGATCCCAGTGAATC-3’ (forward primer)  5’-TCAGTTCAGTTCCAGGTCATACA-3’ (reverse primer)  5’-CCAGCAGTCGTCTTTGTCAC-3’ (forward primer)  5’-CTCTGGGTTGGCACACACTT-3’ (reverse primer)  5’-TACCAGACATCTGAGGAC-3’ (forward primer)  5’-ATTCTTCACTCTCTTGTTGT-3’ (reverse primer)  5’-TGCCCAGCATCATGAAGG-3’ (forward primer)  5’-TCAGGCATTCAGCTTCAGG-3’ (reverse primer)  5’-ATCGCCTACAGACTGCACTC-3’ (forward primer)  5’-GACGGTAACGGACGTAATCAC-3’ (reverse primer)  5’-AACCGAAGTCATAGCCACAC-3’ (forward primer)  5’-GTTGGATTTGTCACTGTTCAGC-3’ (reverse primer)  5’-GCTTGTCTCAACCCCGCATC-3’ (forward primer)  5’-TGGATTTGCCATTTTTCAGCATCTT-3’ (reverse primer)  5’-AGCTGCGTTGCGTTTGTTTAC-3’ (forward primer)  5’-TGGCGAACACTTGCAGATTAC-3’ (reverse primer) |
