# Supplementary material for: Crosstalk between tumor-associated macrophages and tumor cells promotes chemoresistance via CXCL5/PI3K/AKT/mTOR pathway in gastric cancer
Source: Cancer Cell Int. 2022 Sep 23;22:290. doi: 10.1186/s12935-022-02717-5 (PMC9508748; doi:10.1186/s12935-022-02717-5)
Supplement: Supplementary file 3 — Additional file 3: Table S2. Antibody used in the study. [file 12935_2022_2717_MOESM3_ESM.docx]

Supplementary Table S2. Antibody used in the study

| Antibody | Dilution rate | | reference number | company |
| --- | --- | --- | --- | --- |
| P-gp | | 1:1000 | A19093 | Abclonal |
| Bcl-2 | | 1:1000 | A19693 | Abclonal |
| Bax | | 1:1000 | A0207 | Abclonal |
| PI3K | | 1:1000 | A4992 | Abclonal |
| p-PI3K | | 1:1000 | AP0845 | Abclonal |
| AKT | | 1:1000 | 4685 | Cell Signaling Technology |
| p-AKT | | 1:1000 | 4060 | Cell Signaling Technology |
| mTOR | | 1:1000 | 2972 | Cell Signaling Technology |
| p-mTOR | | 1:1000 | 5536 | Cell Signaling Technology |
| GAPDH | | 1:500 | 2118 | Cell Signaling Technology |
| HRP-conjugated Anti-Rabbit IgG | | 1:10000 | 7074 | Cell Signaling Technology |
| FITC-CD11b | | 1:100 | 301330 | BioLegend |
| PE-CD86 | | 1:100 | 374206 | BioLegend |
| APC-CD163 | | 1:100 | 333609 | BioLegend |
| APC-CD206 | | 1:100 | 321110 | BioLegend |
| CD68 | | 1:100 | 26042 | Cell Signaling Technology |
| CD163 | | 1:100 | 25121 | Cell Signaling Technology |
| CD206 | | 1:100 | 24595 | Cell Signaling Technology |
| CXCL5 | | 1:200 | ab271281 | Abcam |
