# Supplementary figures and images for: Crosstalk between tumor-associated macrophages and tumor cells promotes chemoresistance via CXCL5/PI3K/AKT/mTOR pathway in gastric cancer
Source: Cancer Cell Int. 2022 Sep 23;22:290. doi: 10.1186/s12935-022-02717-5 (PMC9508748; doi:10.1186/s12935-022-02717-5)

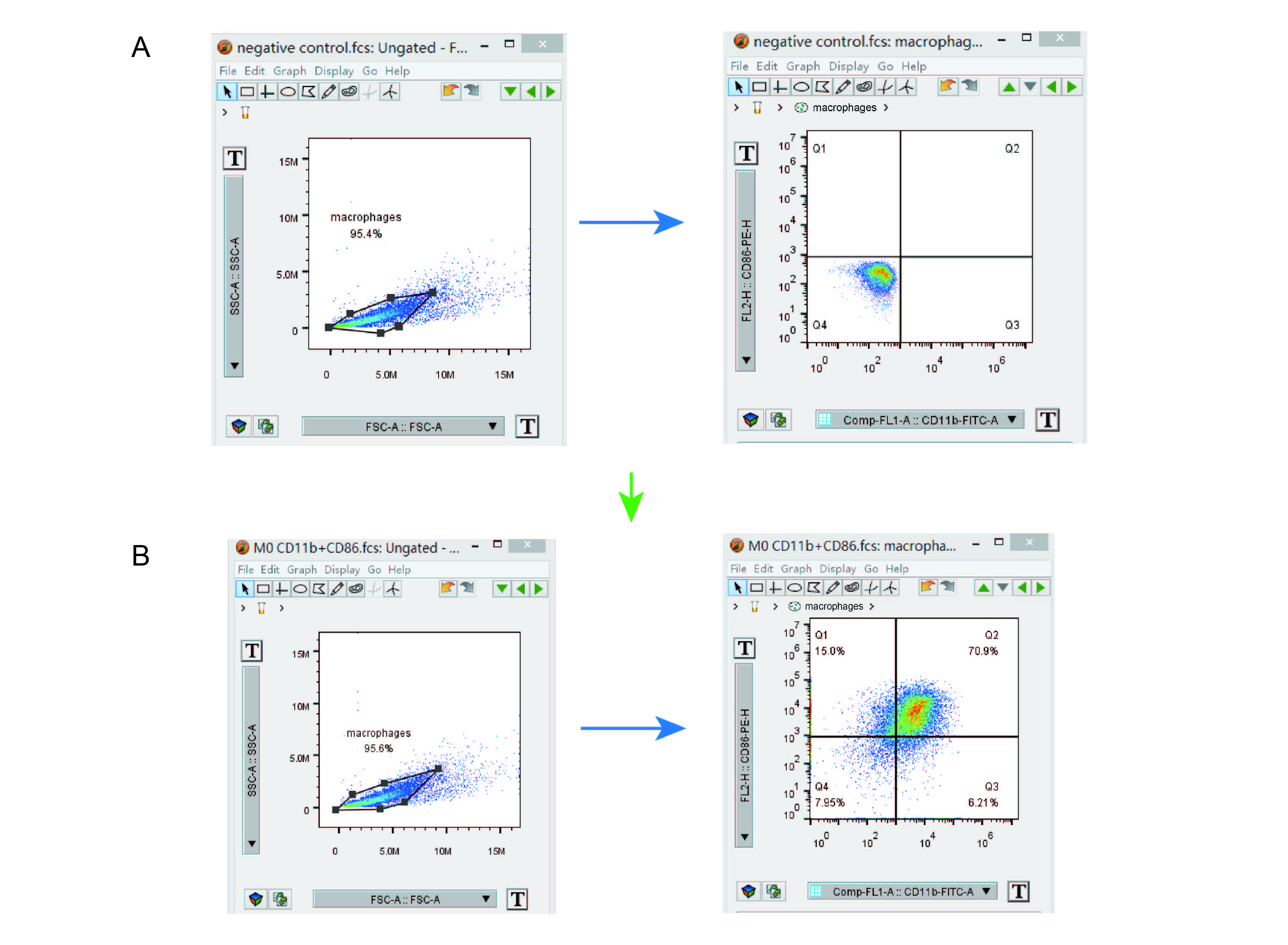

Supplement: Supplementary file 4 — Additional file 4: Figure S1. Gating strategy for flow cytometry. [file 12935_2022_2717_MOESM4_ESM.jpg]

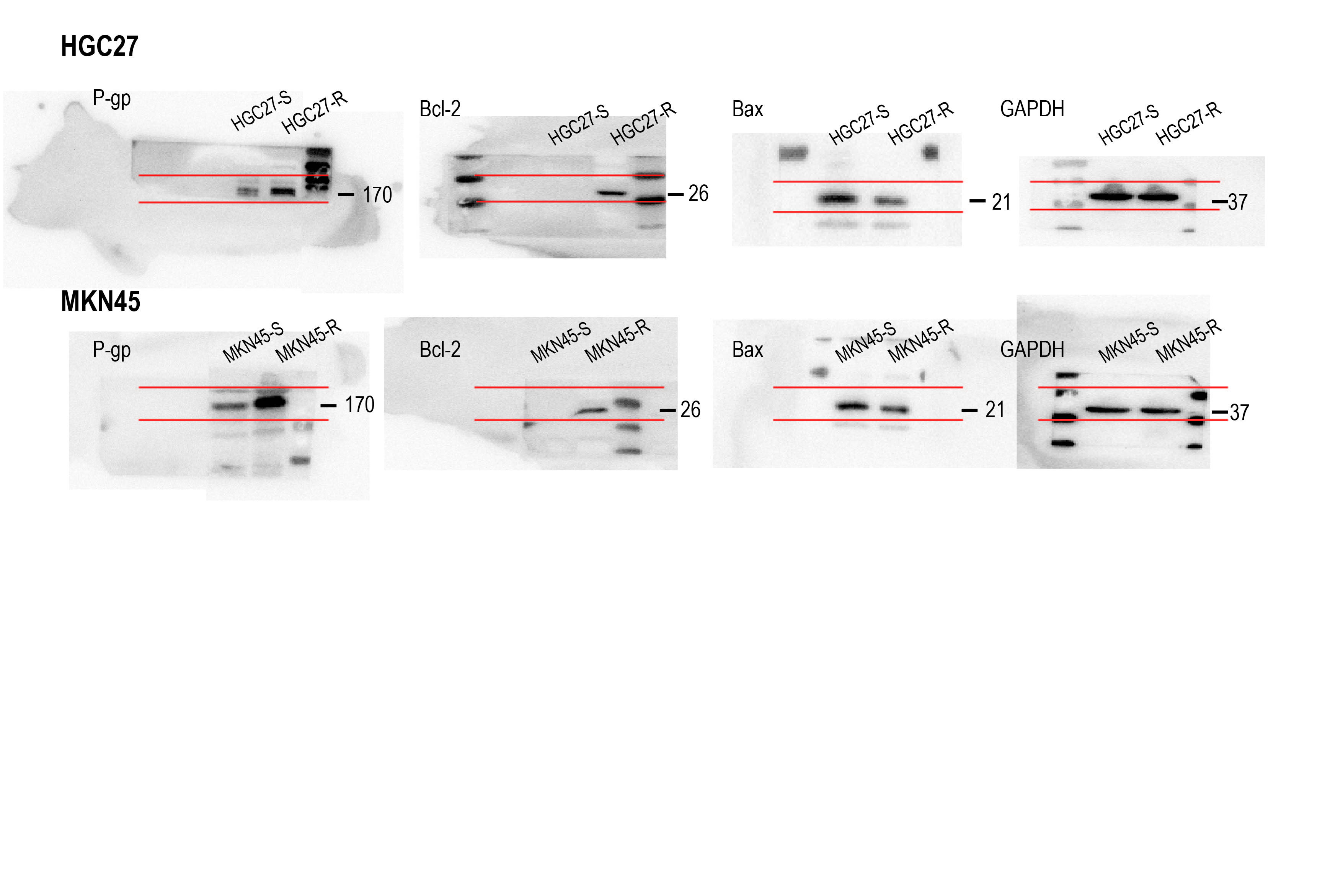

Supplement: Supplementary file 5 — Additional file 5: Figure S2. Uncropped western blot images of Fig. 2. [file 12935_2022_2717_MOESM5_ESM.jpg]

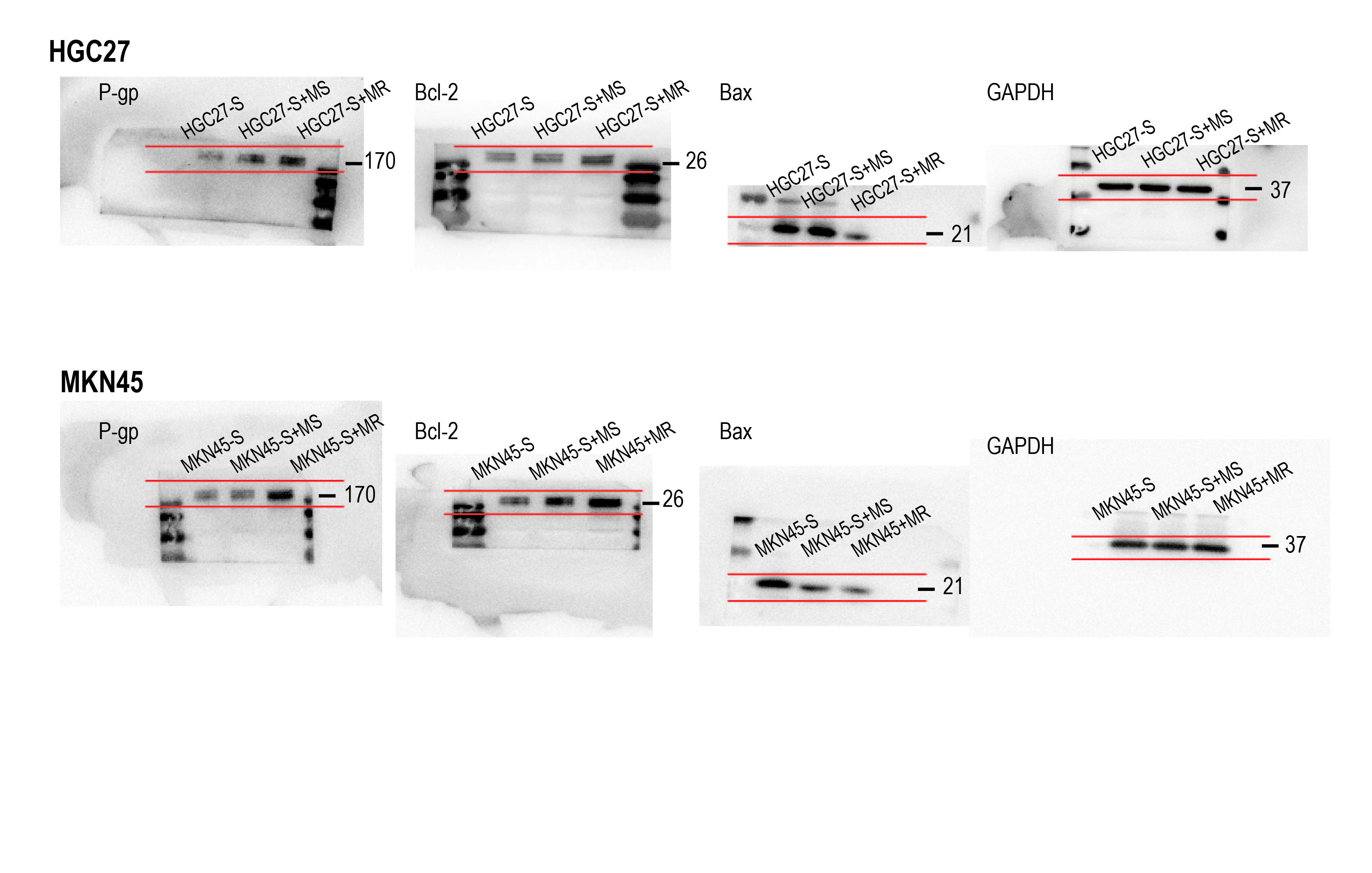

Supplement: Supplementary file 6 — Additional file 6: Figure S3. Uncropped western blot images of Fig. 4. [file 12935_2022_2717_MOESM6_ESM.jpg]

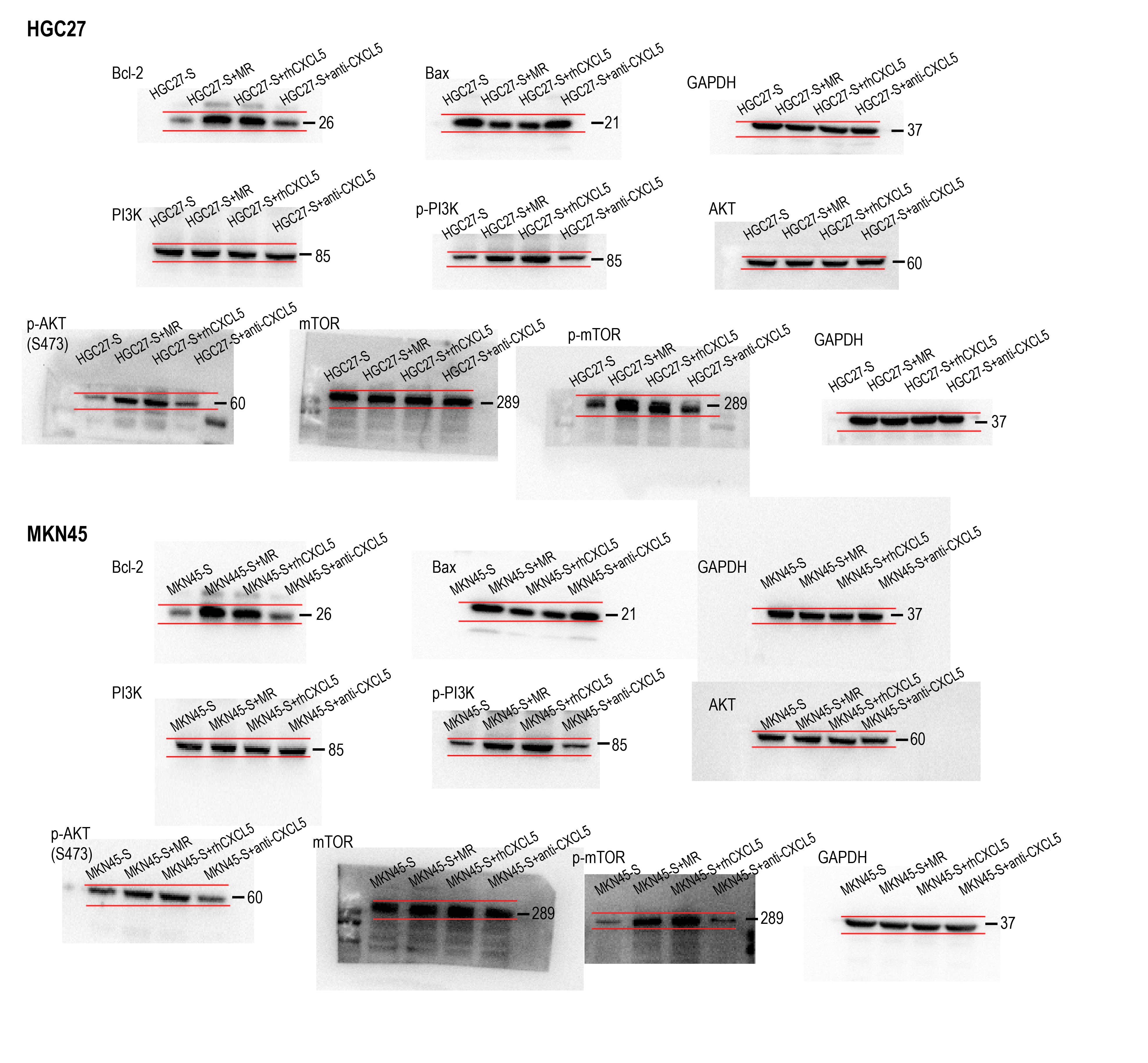

Supplement: Supplementary file 7 — Additional file 7: Figure S4. Uncropped western blot images of Fig. 6. [file 12935_2022_2717_MOESM7_ESM.jpg]
